# Desserts Enriched with a Nanoemulsion Loaded with Vitamin D_3_ and Omega-3 Fatty Acids for Older People

**DOI:** 10.3390/foods13132073

**Published:** 2024-06-29

**Authors:** Natalia Riquelme, Paz Robert, Carla Arancibia

**Affiliations:** 1 Departamento de Ciencia y Tecnología de los Alimentos, Facultad Tecnológica, Universidad de Santiago de Chile (USACH), Obispo Manuel Umaña 050, Estación Central, Santiago 9170201, Chile; natalia.riquelme.h@usach.cl; 2 Departamento de Ciencia de los Alimentos y Tecnología Química, Facultad de Ciencias Químicas y Farmacéuticas, Universidad de Chile, Santos Dumont 964, Independencia, Santiago 8380494, Chile; proberts@uchile.cl

**Keywords:** older people, desserts, enriched nanoemulsion, bioaccessibility, sensory performance

## Abstract

The food industry is challenged to develop nutritious and palatable foods that satisfy older people’s needs. So, this work aimed to study the incorporation of nanoemulsions enriched with vitamin D_3_ and omega-3 fatty acids into two desserts (yogurt and fruit puree), characterizing their nutritional profile, viscosity, and color properties and evaluating their in vitro bioaccessibility and sensory response. The results showed that adding nanoemulsion modified the nutrition profile of desserts due to increasing lipids and calories. The desserts’ physical properties were also affected, with a decrease in viscosity and a lightening of color. Regarding digestion, the enriched desserts presented a low release of free fatty acids (14.8 and 11.4%, respectively). However, fruit puree showed the highest vitamin D_3_ and omega-3 fatty acid in vitro bioaccessibility (48.9 and 70.9%, respectively). In addition, older consumers found this dessert more acceptable than yogurt due to the adequate intensity of its sensory attributes (aroma, flavor, sweetness, and consistency). Therefore, the fruit puree can be enriched with nanoemulsions loaded with vitamin D_3_ and omega-3 fatty acids to improve the bioaccessibility of lipid bioactive compounds and sensory performance, offering a health-enhancing option for older consumers.

## 1. Introduction

Malnutrition has been detected in older people. It has been attributed to psychological, physiological, and environmental factors that affect their food consumption [1]. In addition, older people undergo different changes in sensory perception and present difficulties in the chewing and swallowing process, which cause a decline in food intake [2]. Therefore, developing appropriate food products is crucial for reducing the risk of chronic diseases. Food enrichment with bioactive compounds can be an excellent alternative to relieve some physiological dysfunctions and improve the nutrition of older people.

An innovative alternative for developing enriched foods is using nanoemulsions as carriers of bioactive compounds. Nanoemulsions, such as conventional emulsions, consist of a dispersion of two immiscible liquids (generally oil and water), where one of the phases is dispersed in the other in the form of small droplets (50–200 nm). They can be classified according to the relative spatial distribution of their phases: water in oil (W/O) or oil in water (O/W) [3]. Additionally, nanoemulsions act as delivery systems during digestion due to their reduced droplet size, which facilitates rapid interaction with the biological components of the gastrointestinal system. Furthermore, nanoemulsions may improve the bioaccessibility and stability of the lipid compounds incorporated into them [4]. Several studies have reported that nanoemulsions improve the bioaccessibility of lipid compounds [5,6,7], where these systems behave as excellent carriers of lipid compounds essential for older people’s nutrition, such as ω-3 fatty acids and vitamin D.

ω-3 polyunsaturated fatty acids (eicosapentaenoic acid-EPA and docosahexaenoic acid-DHA) are essential nutrients for older people. Their regular consumption reduces the risk of developing neurodegenerative diseases [8]. EPA and DHA are present in the fat/oil of fish, microalgae, and marine invertebrates; however, many consumers do not like their taste or do not consume these kinds of products. Furthermore, EPA and DHA are sensitive to lipid oxidation, which can deteriorate their nutritional value and sensory properties [9]. Similarly, vitamin D is a micronutrient that plays a vital role in the nutritional status of older people since it is responsible for maintaining the calcium and phosphate levels required for bone mineralization and muscle function [10]. Nevertheless, vitamin D deficiency is expected in the older population because of its limited consumption from food sources and low sun exposure, which decreases its synthesis and conversion into the active form [11]. In this sense, nanoemulsions loaded with bioactive compounds could be incorporated into foods to enrich them since they can improve the physical stability of food during its processing, storage, and digestion [4].

Various approaches have been applied for food fortification with bioactive compounds using nanoemulsions. For example, Almasi et al. [12] prepared a stable flaxseed oil (with omega-3 fatty acids) nanoemulsion that was added to yogurt. Their results showed that nanoemulsions could help increase the solubility, bioavailability, and protection of omega-3 fatty acids. Similarly, Matos et al. [13] nanoencapsulated vitamin D_3_ to enrich coconut yogurts. Findings showed that the nanoemulsion affected the structural characteristics of the samples; however, consumers showed a higher acceptability of the vitamin D-enrich yogurt. Despite this, it is still necessary to develop new soft and moist foods that are easy and safe to swallow, which are characteristics more suitable for older people.

Some studies have also focused on developing food products for older people, using sensory consumer studies to identify the factors influencing their choice and liking to satisfy their sensory needs [14]. According to a previous work about the opinions and perceptions of older people about desserts, they considered fruits and dairy-based desserts that are healthy and palatable alternatives [15], making them suitable for food enrichment. Additionally, these soft-texture foods could reduce the risks of choking and aspiration in older people with swallowing difficulties [16]. Despite this, studies have yet to be conducted to satisfy older people’s sensory and nutritional requirements in developing food adapted to their physiological needs. Therefore, this work aims to study the bioaccessibility of bioactive compounds (vitamin D_3_ and omega-3 fatty acids carried into nanoemulsions) and the sensory performance of two desserts (yogurt and fruit puree). This study expects to obtain enriched desserts to supply older people’s nutritional and sensory needs.

## 2. Materials and Methods

### 2.1. Materials

Oil-in-water nanoemulsions were prepared with vitamin D_3_ (1.0 MIU/g, DSM Co., Basel, Switzerland) and EPA + DHA-rich oil (Fish Oil TG400300, Golden Omega S.A., Arica, Chile) as the lipid phase, soy lecithin (Metarin P-Cargill, Blumos S.A., Santiago, Chile), polysorbate 80 (Food grade, Sigma-Aldrich S.A., Saint Louis, MO, USA) and sucrose esters (Sisterna B.V., Roosendaal, The Netherlands) as emulsifiers, and purified water obtained from an inverse osmosis system (Vigaflow S.A., Santiago, Chile) as the aqueous phase. Strawberry and bananas (for fruit puree) and fat-free yogurt (100 g of product contains 0.4 g of fat, 3.8 g of proteins, and 4.0 g of carbohydrates) (Quillayes Peteroa Spa, Loncoche, Chile) were purchased from the local market and used for the preparation of desserts. For lipid digestion assays, pepsin from porcine gastric mucosa (P6887), pancreatin from porcine pancreas (P1750), bile extract (B8631), and lipase from porcine pancreas (L3126) were obtained from Sigma-Aldrich (Saint Louis, MO, USA). Also, different salts (KCl, KH_2_PO_4_, NaHCO_3_, NaCl, MgCl_2_(H_2_O)_6_, (NH_4_)_2_CO_3_, NaOH, and CaCl_2_(H_2_O)_2_ were used to prepare simulated digestion fluids, which were purchased from Merck (Darmstadt, Germany) and Winkler (Santiago, Chile). Small plastic cups, glass, and spoons (odor-free) were purchased from Vanni S.A. (Santiago, Chile) to serve samples for the sensory study. In addition, crackers (Soda de Costa, Empresas Carozzi S.A., San Bernardo, Chile) and purified water were served to cleanse the palate between samples.

### 2.2. Nanoemulsion and Dessert Preparation

**Figure 1:**
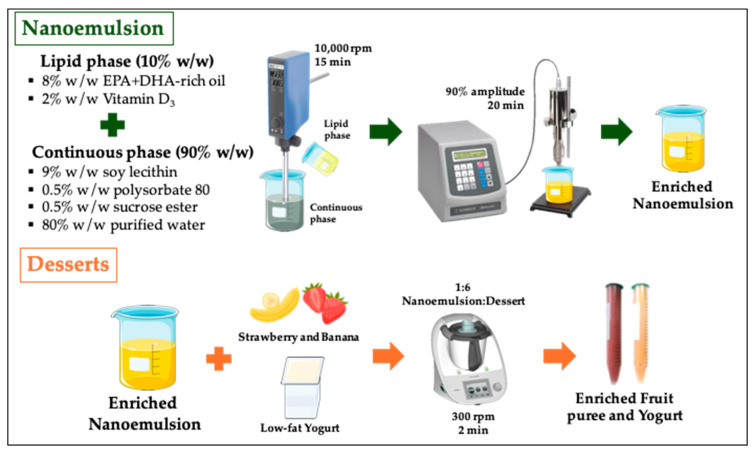
Schematic illustration of the preparation of enriched nanoemulsion and desserts.

The nanoemulsion and dessert preparation are shown in Figure 1. First, the pre-emulsion was prepared by dispersing the 10% *w*/*w* lipid phase (8% *w*/*w* EPA + DHA-rich oil and 2% *w*/*w* vitamin D_3_) into a continuous phase, which consisted of 10% *w*/*w* of emulsifier mixture (9% *w*/*w* soy lecithin, 0.5% *w*/*w* polysorbate 80 and 0.5% *w*/*w* sucrose esters) and 80% *w*/*w* of purified water, which was selected according to previous work [17]. The dispersions were mixed with a high-speed homogenizer (IKA T25, Ultraturrax, Staufen, Germany) at 10,000 rpm and room temperature (25 °C) for 15 min. Then, a homogenized ultrasonic process (VCX500, 20 kHz, 500 W, Sonics, Newtown, CT, USA) was carried out using 90% amplitude, 20 min, and a pulse mode of 15 s on and 10 s off, to decrease the oil droplet size of pre-emulsion. This process was made in an ice bath (10 °C) to prevent the heating of samples. The particle size, polydispersity index, and zeta potential of nanoemulsion were determined using a Zetasizer (Nano ZS, Malvern Instruments, Malvern, UK). Finally, the enriched nanoemulsion (NE) obtained was stored at 4 °C for 24 h before the desserts were prepared.

Raw strawberries and bananas for fruit puree were steamed for 12 min in boiling water (100 °C) and mixed at a 1:1 ratio. Then, the mixture was ground for 2 min at 500 rpm using a kitchen robot (Thermomix TM5, Vorwerk, Wuppertal, Germany) and stored at 4 °C for 24 h. Finally, fruit puree and fat-free commercial yogurt were mixed with the enriched NE (ratio of 1:6, NE:dessert) in a kitchen robot for 2 min at 300 rpm, where each dessert contained ~0.3 g and 1.2 g/100 g product of vitamin D_3_ and EPA + DHA, respectively. Samples were stored at 4 °C for 24 h until their characterization, in vitro digestion, and sensory analysis. At least two batches of each sample were obtained.

### 2.3. Physical Characterization of Desserts

#### 2.3.1. Proximate Composition

The chemical composition of each dessert (fruit puree and yogurt), with and without the enriched NE, was analyzed according to standardized methodologies: moisture [18,19], protein [20], fat [21], ash [22,23], crude fiber [24], no nitrogen extract, and energy [25].

#### 2.3.2. Apparent Viscosity

Viscosity curves were obtained using a rotational rheometer (RheolabQC, Anton Paar, Graz, Austria) equipped with concentric cylinder geometry (CC27 and DG42, Anton Paar, Graz, Austria). Apparent viscosity values were recorded at a shear rate between 1 and 100 s^−1^ for 120 s. The measurements were carried out at 37 °C, controlled by the Peltier system. Samples were allowed to stand for 10 min before assays to stabilize and achieve assay temperature. The apparent viscosity at a shear rate of 50 s^−1^ (η50 s^−1^) was used to compare samples. This shear rate was chosen because it is taken to be the shear rate that occurs during the food swallowing process [26].

#### 2.3.3. Color Properties

The optical properties of enriched nanoemulsion and desserts were determined using a colorimeter (MiniScan XE PLUS, Hunter Associates Laboratory Inc., Reston, VA, USA) under D65 illumination. Colorimeter calibration was performed using the instrument’s black and white calibration plates. For the measure, 15 g of each sample was added to a glass jar, and the CIELab color space parameters (L* = lightness, a* = redness, and b* = greenness) were determined. In addition, the color variation of desserts with or without (control sample) NE was determined using the ΔE2000 equation described by Luo et al. [27].

### 2.4. Lipid Digestion

Enriched nanoemulsion and both desserts (three samples) were subjected to in vitro lipid digestion following the INFOGEST 2.0 protocol for static in vitro gastrointestinal food digestion with some modifications. Static model digestion included three stages (oral, gastric, and intestinal), where specific physiological conditions of older people were considered [28].

*Oral phase:* Twenty-five grams of each sample was mixed with an oral stock solution (3.8% *v*/*v* 0.5 M KCl, 0.9% *v*/*v* 0.5 M KH_2_PO_4_, 1.7% *v*/*v* 1.0 M NaHCO_3_, 0.1% *v*/*v* 0.15 M MgCl_2_(H_2_O)_6_, 0.01% *v*/*v* 0.5 M (NH_4_)_2_CO_3_, 0.02% *v*/*v* 6.0 M HCl, 0.001% *v*/*v* 0.3 M CaCl_2_, and 93.4% *v*/*v* purified water) at a 1:1 ratio. Then, the oral mixture was incubated in a water bath (WNB7, Memmert GmbH+Co, Baviera, Schwabach, Germany) at 37 °C for 2 min at 200 rpm.

*Gastric phase:* Simulated gastric fluid (SGF) was added to the bolus obtained from the oral phase (ratio 1:1). The SGF consisted of a gastric stock solution (1.7% *v*/*v* 0.5 M KCl, 0.2% *v*/*v* 0.5 M KH_2_PO_4_, 3.0% *v*/*v* 1.0 M NaHCO_3_, 3.0% *v*/*v* 2.0 M NaCl, 0.1% *v*/*v* 0.15 M MgCl_2_(H_2_O)_6_, 0.2% *v*/*v* 0.5 M (NH_4_)_2_CO_2_, 0.3% *v*/*v* 6.0 M HCl, 0.001% *v*/*v* 0.3 M CaCl_2_, and 91.5% *v*/*v* purified water) and pepsin (1500 U/mL in the final mixture). The gastric mixture was incubated in a water bath at 37 °C for 90 min at 200 rpm. An automatic titration unit (902 Titrando, Metrohm, Herisau, Switzerland) was used to adjust and control gastric human pH by adding HCL (0.5 M) in three steps: (1) pH 4.0, adding 0.1 mL min^−1^ for 15 min; (2) pH 3.0, adding 0.08 mL min^−1^ for 35 min; and (3) pH 2.0, adding 0.07 mL min^−1^ for 40 min [29].

*Intestinal phase:* The chyme was diluted with simulated intestinal fluid (SIF) at 1:1 after the gastric phase. The SIF was composed of intestinal stock solution (1.7% *v*/*v* 0.5 M KCl, 0.2% *v*/*v* 0.5 M KH_2_PO_4_, 10.5% *v*/*v* 1.0 M NaHCO_3_, 2.4% *v*/*v* 2.0 M NaCl, 0.2% *v*/*v* 0.15 M MgCl_2_(H_2_O)_6_, 0.15% *v*/*v* 6.0 M HCl, 0.01% *v*/*v* 0.3 M CaCl_2_ and 85% *v*/*v* purified water), 10 mM of bile salts, pancreatin (to reach trypsin activity of 50 U/mL), and lipase (1500 U/mL). The intestinal mixture was incubated in a water bath at 37 °C for 180 min at 200 rpm. Lipolysis was monitored using a pH-stat titration unit, which maintained the pH at 7.0, adding 0.5 M NaOH solution. The obtained chyle (raw digest sample) was stored at −18 °C in a centrifuge tube until bioaccessibility analysis.

#### 2.4.1. Fatty Acid Release Kinetics

The amount of free fatty acids (FFAs) released during digestion was calculated from the titration curves using Equation (1):

(1) $$\mathrm{FFA}\;\left(\%\right)=\frac{V_{\mathrm{NaOH}}\times C_{\mathrm{NaOH}}\times M_{\mathrm{lipid}}}{2\times W_{\mathrm{lipid}}}\times 100$$

where V_NaOH_ is the volume of NaOH consumed during intestinal digestion, C_NaOH_ is the concentration of NaOH, M_lipid_ is the molar mass of digestible lipids, and W_lipid_ is the digestible lipid weight in the initial digestion samples.

#### 2.4.2. Bioaccessibility of Bioactive Compounds

After in vitro gastrointestinal digestion, vitamin D_3_ and EPA + DHA bioaccessibility was determined. First, the chyle (digestion sample) was centrifuged (F-1050, Hitachi Ltda., Tokyo, Japan) at 4 °C, 9000 rpm, and 60 min to obtain the micellar fraction. This process separated the samples into two phases, where the clear phase (top of the tube) corresponded to mixed micelles with the solubilized bioactive compounds. Then, the bioactive compounds in the chyle and mixed micelles were extracted and quantified to calculate their bioaccessibility.

The extraction of EPA + DHA from the samples (chyle and micelles) was carried out according to Ng et al. [30] to obtain free fatty acids, which were converted to methyl esters through a derivatization process following the AOAC methodology [31]. The identification and quantification of EPA (C20:5) and DHA (C22:6) were performed following the method proposed by Encina et al. [32], where a GC (7890B, Agilent Technologies, Santa Clara, CA, USA) equipped with a flame ionization detector (FID), a split injection system (Split), and an H-88 fused silica capillary column (0.25 mm × 100 m, 0.20 µm, Agilent Technologies, Santa Clara, CA, USA) were used. The temperature of the injector and detector were programmed at 250 °C. The oven temperature was initially settled at 180 °C for 20 min, and then the temperature was increased at a rate equal to 2 °C/min up to 215 °C, maintaining it for 20 min. In addition, 1 μL of each sample was injected with nitrogen as a carrier gas at a flow rate equal to 1 mL/min.

The extraction and quantification of vitamin D_3_ were carried out following the methodology of Schoener et al. [33], using HPLC (Alliance e2695, Waters, Mildford, MA, USA) equipped with diode-array detection (DAD) (2998 PDA detector, Waters, Mildford, MA, USA). A C18 column (4.6 mm × 250 mm, 0.5 μm, Waters-Ireland) at 25 °C was used as the stationary phase, and methanol:water (95:5) as the mobile phase at a 0.8 mL/min flow rate. The injection volume was 20 μL, and the detection wavelength was 265 nm. Empower 3 software (Waters, Mildford, MA, USA) was used for data analysis.

Finally, the bioaccessibility of bioactive compounds was calculated as follows (Equation (2)):

(2) $$\mathrm{Bioaccessibility}\;\left(\%\right)=\frac{C_{\mathrm{micelle}}}{C_{\mathrm{raw}\;\mathrm{digesta}}}\times 100$$

where C_micelle_ and C_raw digesta_ are the concentrations of vitamin D_3_ or EPA + DHA in the micelle and raw digest after in vitro intestinal digestion.

### 2.5. Sensory Analysis

Sixty older people volunteered from different organizations in the Metropolitan region (Santiago, Chile) to participate in the sensory evaluation of the two desserts (enriched fruit pure and yogurt). The inclusion criteria for recruitment were as follows: people older than 60, living at home, no severe diseases at the study time, and able to communicate for themselves. All participants received and signed their consent to participate in the study according to the protocol approved by the Institutional Ethics Committee from Universidad de Santiago de Chile (No. 483). Samples were presented in a plastic cup (~20 mL) at 10 °C and coded with a three-digit random number. A balanced presentation order (all permutations possible) was used to avoid bias. Crackers and water were also served to cleanse the palate. Data were collected using Compusense software (Academic Consortium Compusense Cloud, Compusense Inc., Guelph, ON, Canada).

Two affective methods were used to evaluate the sensory attribute intensities’ overall liking and suitability. First, consumers were asked about the overall liking of samples (enriched fruit puree and yogurt) using a 7-point structured hedonic scale (1 = Dislike very much, 4 (midpoint) = Neither like nor dislike, and 7 = Like very much) (UNE-EN ISO 11136) [34]. Then, consumers evaluated the ideal level of attribute intensity with a 5-point structured Just About Right (JAR) scale (1 = Too much low, 3 = Just-about-right, 5 = Too much high) for the attributes of aroma, flavor, sourness, sweetness, and consistency. Finally, consumers received a small gift after their complete responses.

### 2.6. Statistical Analysis

The data were analyzed using XLSTAT© 2019 software (Addinsoft, Paris, France). ANOVA (analysis of variance) and Tukey post-test (α = 0.05) were performed for all instrumental data. The liking scores of desserts were analyzed by *t*-test (α = 0.05). Instead, JAR data were analyzed using the frequency distribution percentage of respondents who chose each category of the JAR scale for all sensory attributes studied. Also, a penalty analysis (mean drop) was used to relate JAR data and the overall liking of both samples. To calculate the mean drop, we subtracted the mean overall liking for the JAR group from the mean overall liking of the “Too much low” or “Too much high” categories. Sensory attributes were considered as deviating from the ideal intensity when at least 20% of consumers chose “Too much low” or “Too much high” and had mean drop values > 1.0 [35].

## 3. Results

### 3.1. Nanoemulsion Characteristics

The enriched nanoemulsion with vitamin D_3_ and EPA + DHA-rich oil showed a droplet size of 195 ± 2 nm, a polydispersity index of 0.14, and a zeta potential of −45.6 ± 0.4 mV, indicating good physical stability during storage [3]. This behavior was confirmed after centrifugation, since no creaming or sedimentation was observed. Furthermore, the nanoemulsion showed a Newtonian flow behavior, with viscosity values close to water (~1 mPa·s), which can be attributed to its small droplet size. Finally, the CIELab parameters of enriched NE were L*: 82.41 ± 0.57, a*: 0.55 ± 0.03, and b*: 11.51 ± 0.07, near the milk color.

### 3.2. Dessert Characterization

Table 1 shows the chemical composition of four desserts (with and without NE). Adding enriched NE to both desserts significantly reduced the moisture, protein, and non-nitrogenous extract contents and increased the energy and lipid content. These differences were expected, since the enriched NE contains vitamin D_3_, EPA + DHA-rich oil, soy lecithin, polysorbate 80, and sucrose ester, which alter the dessert’s composition. Similar results were obtained by Razavi et al. [36] in pumpkin puree and Borba et al. [37] in yogurt and ice cream, who argued that incorporating nanoemulsions into foods modified the content of the macronutrients. Accordingly, both enriched desserts presented a fat content lower than 5%, which is characteristic of low-fat products. Therefore, developing low-fat foods and incorporating bioactive compounds may promote older people consumers’ interest in healthier products.

**Figure 2:**
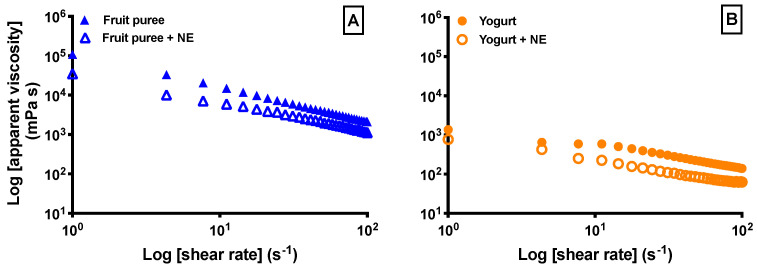
Viscosity curves of fruit purees (**A**) and yogurts (**B**) with or without the enriched nanoemulsion with vitamin D_3_ and EPA + DHA.

Figure 2 shows the apparent viscosity curves of desserts as increasing the shear rate (fruit purees and yogurts, enriched and non-enriched). All samples exhibited pseudoplastic flow behavior since apparent viscosity values decreased with shearing.

Adding the enriched NE significantly reduced (*p* < 0.05) the values of apparent viscosity at 50 s^−1^ of the shear rate in both desserts (65% for yogurt and 54% for fruit puree) (Table 2). Similar results were obtained by Campo et al. [38] and Song and McClements [39], who studied the incorporation of a nanoemulsion with zeaxanthin and turmeric in two different foods: yogurt and salad dressing, respectively. These authors observed that adding nanoemulsions reduces the apparent viscosity of foods between 50 and 74%. On the other hand, according to the International Dysphagia Diet Standardization Initiative (IDDSI), the obtained viscosity values at 50 s^−1^ correspond to categories 2 (mildly thick) and 1 (slightly thick) for fruit purees and yogurts, respectively. This category means that little effort is required to swallow the product, facilitating easy and safe consumption by older people with deglutition problems [40].

**Figure 3:**
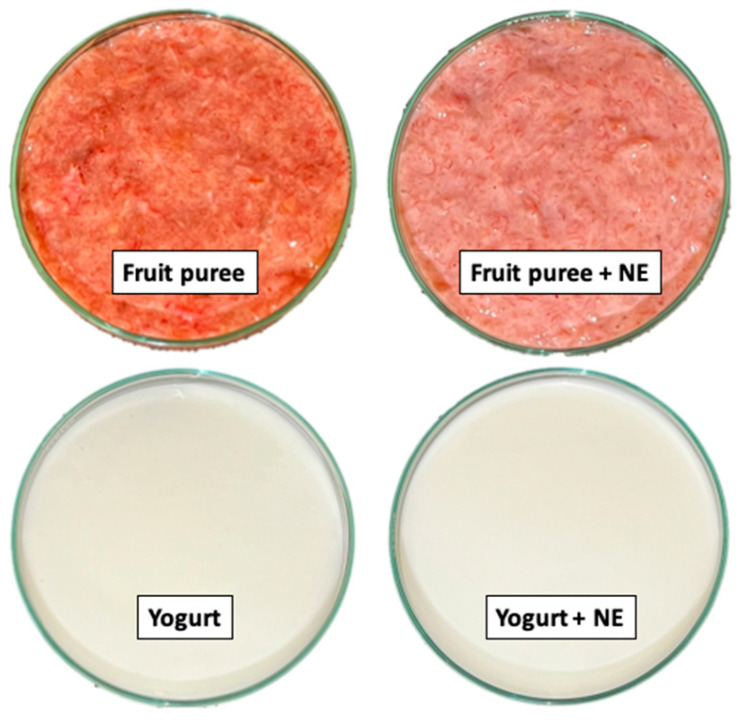
Photographs of the desserts (fruit puree and yogurt) with and without the enriched nanoemulsion (NE) with vitamin D_3_ and EPA + DHA.

Table 2 and Figure 3 show the changes in the color properties of the different desserts fortified with the enriched NE. In general, the incorporation of the NE significantly modified (*p* > 0.05) the color parameters of desserts (Table 2) due to its intrinsic color (nearly the color of milk). For fruit puree, L* (lightness) values significantly increased, while a* (redness) and b* (greenness) decreased, where a ΔE2000 values around 9.0 were found. This result indicates a perception of “much color difference” [41]. Instead, yogurt showed slightly increased color parameters (L*, a*, and b*) with NE addition (*p* > 0.05; Table 2). Additionally, ΔE2000 values ~1.42 were obtained, suggesting a perception of a “slight color difference” [41].

These results show that incorporating the enriched nanoemulsion into the two desserts modified their physical, sensory, and nutritional characteristics. Therefore, these properties must be considered when creating commercial products.

### 3.3. Lipid Digestion

**Figure 4:**
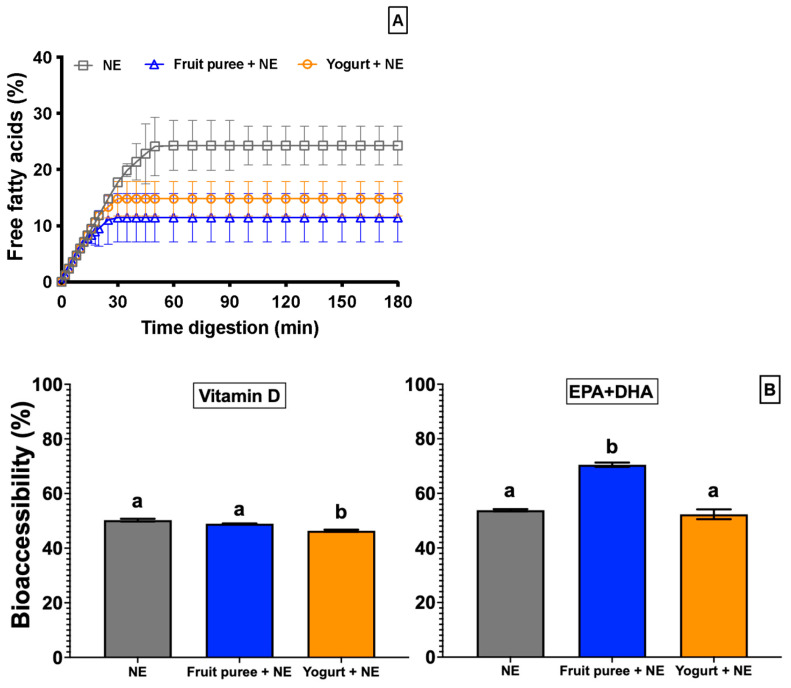
Free fatty acids released during in vitro intestinal digestion (**A**) and the bioaccessibility of vitamin D_3_ and EPA + DHA (**B**) of nanoemulsion (NE) and enriched desserts (fruit puree and yogurt). Different letters indicate significant differences between samples (*p* > 0.05) for the same bioactive compound.

Several studies have demonstrated that older people presented a deteriorated gastrointestinal function, affecting nutrient digestibility [28,42]. Accordingly, enriched nanoemulsion and desserts (three samples) were subjected to an in vitro digestion (protocol INFOGEST 2.0) simulating the physiological conditions of older people: (i) the lowest concentration of digestive enzymes (1500 U/mL of pepsin, 50 U/mL of pancreatin and 1500 U/mL lipase), (ii) modification of the pH of the gastric phase (final pH 3), and (iii) increased intestinal digestion time (180 min) [43]. Figure 4A shows the kinetics of free fatty acid (FFA) release from samples during intestinal digestion, exhibiting similar FFA release profiles. First, a low release of FFA was observed, where %FFA slightly increased during the 20–40 min intestinal digestion. These results differed from those previously reported for emulsioned systems. The %FFA increased more rapidly during the first 10–15 min of intestinal digestion for adults, which is associated with fast lipase adsorption at the oil droplet surface [29]. In our case, the release of FFA during the first minutes was slower due to the simulated conditions for older people, which could trigger low adsorption of the lipases into the oil droplets. This fact may hinder enzymatic penetration into the droplets, limiting enzymatic hydrolysis. After this time, the release of fatty acids remained almost constant, with an equilibrium observed in the percentage of FFA (Figure 4A). According to Inapurapu et al. [5], triglycerides are hydrolyzed to free fatty acids and mono- and diglycerides during intestinal digestion. The authors claim that digestion can inhibit the activity of the lipase because triglycerides can be adsorbed on the surface of oil droplets, displacing the enzyme from the oil–water interface.

The percentage of FFA released from the enriched NE and desserts (yogurt and fruit puree with this NE) was lower than in other studies. Schoener et al. [33], Inapurapu et al. [5], and Teixé-Roig et al. [44] studied the lipid digestion of nanoemulsions with different bioactive compounds, which showed values between 60 and 100% of FFA released versus 10–25% in this work (Figure 4A). These lowest FFA releases from the enriched NE and desserts may be due to different facts: (i) the higher content of free fatty acids was present in EPA + DHA-rich oil (760 mg/g of the lipid phase) used to elaborate the enriched NE, where a decrease in the substrate causes a lower FFA release during digestion; (ii) long-chain polyunsaturated fatty acids (EPA and DHA) show high resistance to in vitro lipolysis because of the presence of a double bond near the carboxyl end of their structure; and (iii) the simulated physiological conditions of older people for in vitro digestion (time and concentration of digestive enzymes) can generate a lower release of FFA in desserts. Older people have a lower capacity to digest and absorb lipids due to deteriorated gastrointestinal system conditions, such as a reduction in the secretion of enzymes, changes in the composition of luminal electrolytes, and a decrease in motility, among others [28]. Therefore, changes in the digestive fluids to simulate older people’s digestion may hinder an adequate digestion of lipid nutrients.

Regarding the final extent of fatty acids released, the enriched NE presented the highest %FFA (26.9%) compared to the two studied desserts (11.4% and 14.78% for fruit puree and yogurt, respectively) (Figure 4A). These differences in %FFA among desserts and enriched NE were expected, since food is a more complex system than a simple oil-in-water emulsion due to its composition and structure. Therefore, we hypothesize that the stability of the food structure and the interaction of lipids with other components (proteins and polysaccharides) during gastrointestinal digestion could decrease lipid digestion. In the case of fruit puree, dietary fibers may alter lipid digestion due to two main mechanisms: (i) the reduction in enzymatic activity given the interaction between fibers and lipases [45] and (ii) the adsorption of the fibers at the oil/water interface, preventing that the lipase reaches the triglycerides for their hydrolysis [46]. In the case of yogurt, the reduction in %FFA can be due to the coagulating milk proteins because of gastric conditions (low pH, high ionic strength, and the presence of pepsin). In this case, the oil drops from the enriched NE might be trapped in this protein matrix, limiting their accessibility to lipase [47]. Furthermore, the release of FFA may also be affected by the formation and precipitation of insoluble complexes between calcium and the long-chain fatty acids of triglycerides (calcium soaps), which accumulate on the droplets’ surface and instead inhibit lipase activity [48].

Finally, these results demonstrate that simulated gastrointestinal conditions for older people and complex food structures decrease lipid digestion. This fact may affect the bioaccessibility of lipid bioactive compounds, including vitamin D_3_ and EPA + DHA, which are essential for older people’s nutrition.

### 3.4. Bioaccessibility

The bioaccessibility of vitamin D_3_ and EPA + DHA from enriched NE and desserts after the in vitro digestion was also determined. Figure 4B shows that all samples presented values of bioaccessibility of vitamin D_3_ close to 50%. These values demonstrate nanoemulsions’ effectiveness in maintaining vitamin D_3_ stability under gastric conditions, protecting it from pH, ionic strength, and shearing. These results agree with those reported by other studies, which obtained percentages of bioaccessibility of vitamin D_3_ between 40 and 60% [33,49]. Enriched yogurt presented slightly lower bioaccessibility values (46.3 ± 0.4%) than fruit puree (49.0 ± 0.1%). This difference may be related to the formation of calcium–triglyceride complexes in yogurt during digestion, affecting mixed micelle formation [48]. In contrast, incorporating the enriched NE into the fruit puree did not affect the bioaccessibility of vitamin D_3_ since no significant differences (*p* > 0.05) were found between these samples (Figure 4B). This result can be attributed to the structural stability of the fruit puree in gastrointestinal conditions since the vitamin D_3_ remains solubilized in the small oil droplets. Thus, the chemical stability of vitamin D_3_ is maintained during the oral and gastric phases. Then, it is released from the chyme and solubilized in the mixed micelles in the small intestine.

The bioaccessibility of EPA + DHA was significantly (*p* < 0.05) higher in fruit puree (70.0 ± 1.5%) than in the enriched NE and yogurt (53.8 ± 0.4% and 52.3 ± 1.8%, respectively) (Figure 4B). According to these results, the dietary fiber in the fruit puree promotes a protective network around the oil droplets that helps maintain their size during the oral and gastric phases [29]. In this sense, the smaller oil droplets maintain their large surface area for lipase adsorption at the interface during the intestinal phase, improving the bioaccessibility of EPA + DHA [44].

Finally, incorporating vitamin D_3_ and EPA + DHA-carried nanoemulsions into the two desserts can increase the bioaccessibility of these bioactive compounds. Consequently, the fruit puree improves the bioaccessibility of vitamin D and EPA + DHA carried into NE after the in vitro digestion. These results may suggest that nanoemulsions may be a valuable tool to modulate the bioaccessibility of essential lipid micronutrients for the nutrition of older people.

### 3.5. Consumer Acceptability and Sensory Perception

Food consumption should be a pleasing sensory experience for older people [50]. Hedonic and Just About Right (JAR) scales were used to identify which sensory attributes significantly influence dessert liking (enriched yogurt and fruit puree).

Sensory analysis was carried out by 60 older people (63% female and 37% male; 52% 60–69 years old, 32% 70–79 years old, and 17% >80 years old), who showed good acceptability (based on a 7-point hedonic scale) of desserts and found significant differences (*p* < 0.05) between samples. The fruit puree was evaluated with a score of 6.02 ± 1.21, indicating that older people “like it”, while yogurt presented a lower score (5.12 ± 1.77), evidencing that they “liked slightly” this sample. These results were anticipated. Soft, smooth, and moist food products are popular among older people since these characteristics are associated with a lower risk of choking or easy deglutition. Furthermore, according to a previous study, these products are considered healthy and liked by them [15].

**Figure 5:**
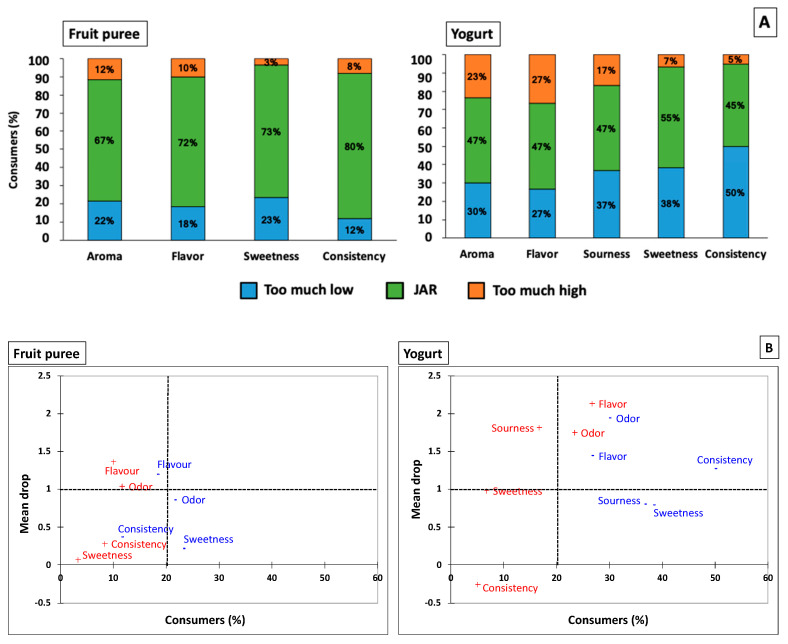
Percentage of respondents responding with the “too much low”, just-about-right (“JAR”), and “too much high” levels of the attributes aroma, flavor, sourness, sweetness, and consistency (**A**); and mean drop plots (**B**). Mean drop: the difference between the mean overall liking score for the JAR group and the mean overall liking score of the “too much and too much high” or “too low and too much low” categories of the 5-point scale. The red words correspond to the “too much and too much high” categories and the blue ones to the “too low and too much low” of the JAR scale. The dashed line represents the boundary of 20% of consumers and mean drop values lower than 1.

JAR scales were used to identify if the sensory attributes were “Just about right” or if it was necessary to increase or decrease their intensity [35]. The JAR test results can be seen in Figure 5A, where a high percentage of the older people perceived that the fruit puree presented an adequate intensity of aroma (67%), flavor (72%), sweetness (73%), and consistency (80%), where this result was considered as an “ideal” product. However, this behavior was not observed in the yogurt, since the aroma (47%), flavor (47%), and sourness (47%) intensities were not perceived as having adequate levels by nearly half of the older consumers. In addition, the consistency was perceived as “much too low consistency” by 50% of consumers (Figure 5A), which can be negative for a safe swallowing process.

A penalty analysis was conducted to determine if the sensory attributes affected the acceptability of samples. Figure 5B shows the results for enriched fruit puree and yogurt, where the *X*-axis corresponds to the percentage of consumers who responded to the category “too much/too low” and “too much/too high”, and the *Y*-axis represents the values of the mean drop for the different attributes. It is noteworthy that these attributes perceived as “low” or “high” intensities by at least 20% of consumers (right side of figures) cannot be selectable for improvement [35]. In addition, a mean drop > 1.0 can be used to select attributes that should be modified or reformulated, according to Rothman [51]. In the case of fruit puree, all attributes were perceived with JAR intensities by >65% of older people (Figure 5B). According to these results, older people conferred high acceptability to fruit puree (~6.0 on a 7-point scale) due to their sensory attributes, which were perceived with optimal intensity. These results agree with a previous work, where older people prefer fruit-based products due to their nutritional characteristics (low in fat and calories and a good source of vitamins and minerals) and sensory and hedonic properties (sweet, soft, refreshment, and “I like it”) [15].

The yogurt’s consistency, sourness, and sweetness were perceived as “too little” by at least 30% of consumers and presented mean drop values < 1.0 (Figure 5B), which negatively affected their acceptability. These results may be due to the poor sensory characteristics of light yogurt since the low-fat content affects its viscosity and flavor and, consequently, its liking. According to Ares et al. [52], low-fat yogurts’ most important sensory defect is their low viscosity. Consumers expect to find plain yogurt texture characteristics in these yogurts, such as creamy and thick. Furthermore, the deficiencies in sourness and sweetness attributes may be due to the lower physiological perception of older people. In addition, yogurt’s flavor and aroma attributes showed the highest mean drop values (>1.4) (Figure 5B); however, there was no consensus among older people regarding the intensity of these attributes since yogurt was perceived as “too much/too low” by the same percentage of consumers.

Finally, several studies have proposed that the desirable texture for food products for older people should be viscous, smooth, creamier, easy to swallow, and non-sticky or grainy [52,53]. Therefore, fruit puree may be an excellent dessert for delivering lipid nutrients that are essential to the health and wellness of older people, whose sensory characteristics are adequate for their sensory requirements.

## 4. Conclusions

In this study, two desserts (yogurt and fruit puree) were enriched by adding a nanoemulsion (NE) loaded with vitamin D_3_ and EPA + DHA-rich oil, which improves their nutritional profile by adding healthy lipids. Furthermore, the addition of NE affected and modified their physical properties, which was more evident in fruit puree. The percentage of fatty acids released was lower than the enriched NE. However, both desserts presented good bioaccessibility (close to 50%) of the bioactive compounds, although fruit puree obtained the best bioaccessibility due to its dietary fiber that protected the bioactive compounds during oral and gastric digestion. In addition, fruit puree showed good acceptability by older people. Meanwhile, the yogurt presented an inadequate intensity of aroma, flavor, and consistency attributes, decreasing its acceptability. Finally, the design of dessert for older people could be carried out by considering two points of view, engineering and sensory approach, through the development of more bioaccessible nanoemulsions enriched with vitamin D_3_ and EPA + DHA and their subsequent incorporation into sensory-adapted dessert such as fruit puree for this group of the population.

## Figures and Tables

**Table 1:** Proximate analysis (g/100 g) of desserts (fruit puree and yogurt) with and without the nanoemulsion enriched with vitamin D_3_ and EPA + DHA.

Parameter	Puree Fruit	Puree Fruit + NE	Yogurt	Yogurt + NE
**Moisture**	84.72 ± 0.08 ^c^	84.17 ± 0.02 ^d^	86.52 ± 0.04 ^a^	85.60 ± 0.02 ^b^
**Proteins (%N × 6.38)**	0.74 ± 0.01 ^c^	0.74 ± 0.02 ^c^	4.65 ± 0.08 ^a^	4.06 ± 0.04 ^b^
**Lipids**	ND	2.74 ± 0.11 ^a^	ND	2.43 ± 0.07 ^a^
**Ashes**	0.59 ± 0.02 ^b^	0.58 ± 0.02 ^b^	1.06 ± 0.01 ^a^	1.03 ± 0.01 ^a^
**Crude fiber**	0.63 ± 0.01 ^a^	0.65 ± 0.03 ^a^	ND	ND
**Non-nitrogenous extract**	13.32 ± 0.07 ^a^	11.11 ± 0.12 ^b^	7.78 ± 0.03 ^c^	6.87 ± 0.01 ^d^
**Energy (Kcal/100 g)**	56.24 ± 0.24 ^c^	72.08 ± 0.43 ^a^	49.69 ± 0.21 ^d^	65.59 ± 0.40 ^b^

**Note:** NE: enriched nanoemulsion, ND: not detected. Different letters indicate significant differences between samples (*p* > 0.05) in the same row.

**Table 2:** Characteristics of desserts (fruit puree and yogurt) with and without the enriched nanoemulsion with vitamin D_3_ and EPA + DHA.

		Fruit Puree	Fruit Puree + NE	Yogurt	Yogurt + NE
**Rheological parameters**	**η50 s** **^−1^** **(mPa·s)**	4001.6 ± 218.6 ^a^	2161.8 ± 425.7 ^b^	225.3 ± 7.8 ^c^	79.0 ± 8.1 ^d^
**Color parameters**	L*	35.83 ± 0.16 ^c^	45.71 ± 0.97 ^b^	85.52 ± 0.13 ^a^	84.43 ± 0.27 ^a^
	a*	17.22 ± 0.55 ^a^	14.76 ± 0.57 ^b^	−2.54 ± 0.06 ^c^	−1.70 ± 0.05 ^d^
	b*	7.58 ± 0.25 ^b^	5.64 ± 0.12 ^c^	9.68 ± 0.21 ^a^	10.29 ± 0.04 ^a^
	**ΔE2000**	9.01 ± 0.82 ^a^		1.42 ± 0.13 ^b^	

**Note:** NE: enriched nanoemulsion. ΔE2000: color difference. Different letters indicate significant differences between samples (*p* > 0.05) in the same row.

## Data Availability

The raw data supporting the conclusions of this article will be made available by the authors on request.
